# Probing ADP Induced Aggregation Kinetics During Platelet-Nanoparticle Interactions: Functional Dynamics Analysis to Rationalize Safety and Benefits

**DOI:** 10.3389/fbioe.2019.00163

**Published:** 2019-07-18

**Authors:** Souvik K. Bandyopadhyay, Mohammad Azharuddin, Anjan K. Dasgupta, Bhaswati Ganguli, Sugata SenRoy, Hirak K. Patra, Suryyani Deb

**Affiliations:** ^1^GlaxoSmithKline Asia Pvt. Ltd., Bangalore, India; ^2^Department of Clinical and Experimental Medicine (IKE), Linköping University, Linköping, Sweden; ^3^Department of Biochemistry, University of Calcutta, Kolkata, India; ^4^Department of Statistics, University of Calcutta, Kolkata, India; ^5^Wolfson College, University of Cambridge, Cambridge, United Kingdom; ^6^Department of Chemical Engineering and Biotechnology, University of Cambridge, Cambridge, United Kingdom; ^7^Department of Biotechnology, Maulana Abul Kalam Azad University of Technology, Kolkata, India

**Keywords:** platelet aggregation, functional data analysis, ADP, nanoparticle, Bi-stable system

## Abstract

Platelets, one of the most sensitive blood cells, can be activated by a range of external and internal stimuli including physical, chemical, physiological, and/or non-physiological agents. Platelets need to respond promptly during injury to maintain blood hemostasis. The time profile of platelet aggregation is very complex, especially in the presence of the agonist adenosine 5′-diphosphate (ADP), and it is difficult to probe such complexity using traditional linear dose response models. In the present study, we explored functional analysis techniques to characterize the pattern of platelet aggregation over time in response to nanoparticle induced perturbations. This has obviated the need to represent the pattern of aggregation by a single summary measure and allowed us to treat the entire aggregation profile over time, as the response. The modeling was performed in a flexible manner, without any imposition of shape restrictions on the curve, allowing smooth platelet aggregation over time. The use of a probabilistic framework not only allowed statistical prediction and inference of the aggregation signatures, but also provided a novel method for the estimation of higher order derivatives of the curve, thereby allowing plausible estimation of the extent and rate of platelet aggregation kinetics over time. In the present study, we focused on the estimated first derivative of the curve, obtained from the platelet optical aggregometric profile over time and used it to discern the underlying kinetics as well as to study the effects of ADP dosage and perturbation with gold nanoparticles. In addition, our method allowed the quantification of the extent of inter-individual signature variations. Our findings indicated several hidden features and showed a mixture of zero and first order kinetics interrupted by a metastable zero order ADP dose dependent process. In addition, we showed that the two first order kinetic constants were ADP dependent. However, we were able to perturb the overall kinetic pattern using gold nanoparticles, which resulted in autocatalytic aggregation with a higher aggregate mass and which facilitated the aggregation rate.

## Introduction

Platelets are very small circulating blood cells, that play pivotal roles in hemostasis and blood coagulation. Upon blood vessel wall injury, platelets are activated by collagen and thrombin, which triggers a platelet aggregation cascade and initiates hemostasis (Packham, 1994). Platelets release auto regulating agonists such as ADP, which activates neighboring resting platelets and thus induces aggregates (Murugappa and Kunapuli, 2006). Among all agonists, ADP induced platelet aggregation received particular attention from researchers and clinician owing to the intriguing involvement of two types of platelet ADP receptors; the high affinity P2Y1 and low affinity P2Y12 receptors, in ADP mediated platelet aggregation (Jagroop et al., 2003). The low affinity ADP receptor P2Y12 is known to be a specific target of anti-platelet drugs such as *ticagrelor* and *clopidogrel*; major therapeutic agents for acute coronary syndrome (ACS) (Dorsam and Kunapuli, 2004; Bednar et al., 2016). Interestingly, low dose ADP can activate P2Y1 to form an unstable micro-aggregate that dissociates with time (known as de-aggregation). Beyond a critical dose, ADP activates P2Y12 resulting in a sharp phase transition from de-aggregation to aggregation. Under disease conditions or in the presence of antiplatelet drugs, this pattern of aggregation, de-aggregation undergoes changes (Maayani et al., 2001). Although the ADP induces aggregation, the de-aggregation phenomenon has been widely known for decades, while the kinetics involved have not been well-studied or explored in depth for several reasons. For example, platelet aggregation is an inter-cellular phenomenon instead of an intra-cellular phenomenon, and has a faster kinetic rate than other physiological events such as cell division, apoptosis etc. Again, it requires the presence of permissive plasma physiological conditions to ensue, therefore, it is hard to monitor any inter-platelet modulation during aggregation under laboratory conditions. A deeper understanding of ADP induced platelet aggregation is a pre-requisite for a better interpretation of the effects during disease conditions and/or during estimation of the drug efficacy on platelet function. Insights in this area would help clinicians in treating patients according to the individual's drug response profile.

Platelets are required for an immediate response to injury, and so they are naturally very sensitive to different types of physiological or non-physiological stimuli. Therefore, nanoparticle-platelet interactions received immense attention for rationalizing both the safety and benefits of using nanoscale materials in the healthcare system. A number of metallic, polymeric, carbon based, and organic nanoforms were tested for their effectiveness as modulators of platelet functions. While nanoparticles have been reported to have pro-thrombotic (aggregator) effects in several studies (Radomski et al., 2005; Deb et al., 2007, 2011, 2012; Oberdörster et al., 2007; Akchurin et al., 2008; Geys et al., 2008), inhibition of platelet aggregation by nanoforms has been reported in very few studies (Shrivastava et al., 2009).

Therefore, pharmaceutically, it is extremely important to explore the effects of nanoparticles on platelet functions. This will not only contribute to a better formulation of nanomedicines but will also reduce the hazardous side effects and limit the chances of unwanted nanoparticle pollution. At this point, it is also important to note that nanoparticle polluted air can easily evade the alveolar spaces of the lung through inhalation and can easily reach the circulation, where their main target is the platelets (Yamawaki and Iwai, 2006; Chen et al., 2008). Our group has explored the complexity of nanoparticle induced alterations in platelet function well and has established that metallic nanoparticles can affect ADP induced platelet activation (Deb et al., 2007, 2011, 2012).

In all our earlier reports as well as those by others, (Deb et al., 2007, 2011, 2012) the data analysis has been performed (ignoring individual signature) in order to obtain a single summary value such as the peak aggregation rate, followed by conventional statistics on this dataset. In the present investigation, we used a novel approach, where we have considered the entire platelet aggregation signature as the outcome and which has been facilitated by the use of a recent class of methods known as Functional Data Analysis (FDA) (Ramsay and Silverman, 2005). FDA constitutes a suite of methods for modeling a response curve, thus enabling us to understand the evolution of the signature over time. Furthermore, we have estimated the derivatives that enabled us to interpret the underlying kinetics, while functional regression methods enabled us to study the dependence of these on the administered drug and its dosage, as well as to quantify the extent of inter-individual signature variations.

## Materials and Methods

### Experimental Data

We have used data sets from two different experiments performed using optical aggregometry. In the first set, platelet aggregation kinetics was studied in presence of ADP while in the second set, alterations in ADP induced platelet kinetics were measured in presence of the gold nanoparticles (AuNPs). For each individual experiment, 10 ml of blood was collected from normal healthy individuals, with their prior consent, and divided into two halves. The first half of 9 ml of blood was mixed with 3.2% tri-sodium citrate as an anticoagulant (final ratio 9:1, whole blood: citrate) and was incubated at room temperature for 5 min. This citrated blood was then centrifuged at 200 × g for 10 min to obtain platelet rich plasma (PRP) from the supernatants. Platelet poor plasma (PPP) was obtained by centrifugation of the PRP at 1,500 × g for 10 min and PPP served as a blank during aggregation study. From the remaining 1 ml of the blood, 0.5 ml blood was mixed with ethylenediaminetetraacetic acid (EDTA) for counting the platelet numbers using an automated cell counter (SysmexSF3000), to confirm that platelet counts were in the normal range (150,000–450,000/μL). The effects of ADP and AuNP on platelet aggregation were measured using standard optical aggregometry techniques, flow cytometry, and optical microscopy. ADP concentrations were chosen according to an individual response owing to large inter-individual variations, from very low to high (to enable us for the detection of de-aggregation to aggregation). The ChronoLog instrument (Model 530BS) was used to study platelet aggregation, and a BD Facs Calibur flow cytometer was used to study platelet activation and a Nikon eclipse Ti microscope was used to observe and image the platelets aggregates.

To assess ADP induced platelet aggregation kinetics, a similar as above PRP was prepared from the blood of a healthy normal individual with normal platelet counts and was treated with different ADP (Chrono-log corp, Havertown, PA) concentrations (varying from low to high). The platelet aggregation kinetics was measured for approximately 6–7 min. To determine the effects of nanoparticle perturbation on platelet aggregation, 40 μM AuNPs with hydrodynamic diameters of 20 nm were used as optimum candidate metallic nanoparticles to assess their effects on platelet function. We have previously reported that a specific nanosystem can induce optimum pro-aggregatory effect on platelets (Deb et al., 2011). We prepared the AuNPs by citrate reduction of HAuCL_4_ (Arora Matthey Limited, Kolkata, India) as we have previously reported (Deb et al., 2007, 2011, 2012). A detailed description of AuNP preparation, microscopy, optical aggregometry and flow cytometric detection of AuNP induced platelet activation is provided in the Supplementary Materials.

### Statistical Analysis

Let *y*_*i*_(*t*) denote the percentage of platelet aggregation observed at time t for the ith subject, where *t* = (*t*_1_, *t*_2_, …, *t*_*L*_) denotes the set of L times at which observations are recorded for each subject; *i* = 1, 2, …, *n*. Functional data analysis assumes a model of the form,

(1) $$\begin{array}{l} y_{i}\left(t\right)=f\left(t\right)+\;\epsilon_{it} \end{array}$$

where, *f*(*t*) is an underlying unknown signal which is assumed to be smooth and ϵ_*it*_ denotes random noise. Note that no additional assumptions are made about the true signal except that it is smooth i.e., second order derivatives exist and are continuous.

We shall first represent *f*(*t*) using a truncated polynomial spline basis (Ruppert et al., 2003). These are a set of flexible basis functions which allow the underlying function *f*(*t*) to depart from linearity, without imposing any restrictions on its shape. More specifically, we assume *f*(*t*) of the form

(2) $$\begin{array}{l} f\left(t\right)=\;\overset{p}{\underset{j=0}{\sum }}\beta_{j}t^{j}+\;\overset{K}{\underset{k=1}{\sum }}u_{k}{\left(t-\;\kappa_{k}\right)}_{+}^{p}, \end{array}$$

Where, κ_1_ < κ_2_ < … < κ_*K*_ are K knots and ${\left(\beta_{0},\;\ldots ,\;\beta_{p},u_{1},\;\ldots ,\;u_{K}\right)}^{\prime }$ are the unknown regression coefficients. Here we have used p^th^ degree truncated basis function defined by

(3) $${\left(t-\;\kappa_{k}\right)}_{+}^{p}=\;\{\begin{array}{c} {\left(t-\;\kappa_{k}\right)}^{p},\;if\left(t-\;\kappa_{k}\right)>0\\ 0,\;if\;\left(t-\;\kappa_{k}\right)<0 \end{array}$$

The degree of the piecewise polynomials is set at 5 to ensure that the estimated functions as well as its estimated derivative are smooth curves (Ruppert et al., 2003, 2009). Furthermore, we assumed that the coefficients of the basic functions are random variables which are normally distributed with zero mean and variance ${\;\sigma }_{u}^{2}$. Such an assumption can be shown to impose a smoothness on the shape of the estimated *f*(*t*) by effectively imposing a penalty on the contribution of each basis function and leads to what is called the Linear Mixed Model (LMM) (Ruppert et al., 2003) formulation of penalized splines, a well-studied model in statistical literature.

The advantage of such a model formulation is that it allows us to use standard Best Linear Unbiased Prediction (BLUP) based inference (Robinson, 1991) and confidence estimation, as well as to implement our analysis using standard software packages developed to fit an LMM. An interesting consequence of our model is the possibility to use BLUPs to derive an estimator of the first and higher order derivatives of the curve, *f*(*t*).

The above model can also be easily extended to include covariate information and/or additional individual heterogeneity in responses, which is not explained by the regression model. For example, a simple model incorporating information on the dose for the first data set can be written in the form of;

(4) $$\begin{array}{l} y_{dj}=f_{1}\left(t_{j}\right)+\;\overset{7}{\underset{d=2}{\sum }}I_{d}f_{d}\left(t_{j}\right)+\epsilon_{dj} \end{array}$$

where *f*_*d*_(*t*) denotes the model based mean platelet aggregation at time *t* in response to dose *d* of a drug and *I*_*d*_'s are indicators that a subject has been assigned to the corresponding dose, *d*. As before, the functions, *f*_*d*_(*t*) are unknown and assumed to be smooth, modeled using a fifth-degree truncated spline.

We could have extended the modeling strategy by introducing another covariate which is an indicator of whether the blood sample received nanoparticles and it can model the effect of nanoparticles as a smooth function of time. However, we take a simpler course where we use the previous model representation of Equation (4) i.e.,

(5) $$\begin{array}{l} y_{dj}=f_{1}\left(t_{j}\right)+\;\overset{10}{\underset{d=2}{\sum }}I_{d}f_{d}\left(t_{j}\right)+\epsilon_{dj}, \end{array}$$

where *f*_*d*_(*t*) is defined as before. Here *d* = 1, 2, …., 5 corresponding to five doses of ADP while *d* = 6, 7, …., 10 corresponds to the same dose of ADP along with the addition of nanoparticles. An estimate (denoted by ^∧^) of the effect of nanoparticles for the *s*^th^(*s* = 1, 2, …, 5) dose level (*ĥ*_*s*_(*t*_*j*_)) can be obtained by subtracting the two estimated curves i.e. ${ĥ}_{s}\left(t_{j}\right)={\hat{l}}_{\left(5+s\right)}\left(t_{j}\right)-\;{\hat{l}}_{s}\left(t_{j}\right);s=1,2,\;\ldots ,\;5$ where ${{\hat{l}}_{d}\left(t_{j}\right)=\;\hat{f}}_{1}\left(t_{j}\right)+\;{\hat{f}}_{d}\left(t_{j}\right),\;d=2,\ldots .,10.$ The overall estimate of the effect of the nanoparticle is obtained by taking the mean of *ĥ*_*s*_(*t*_*j*_); *s* = 1, 2, …, 5.

### Differential Equation Models

Our FDA model formulation allows exploring the dynamic system, using the fitted first and second derivatives. The advantage of FDA is that we can map the variable (y) with its velocity ($\frac{dy}{dt}$) using a linear function and then explore the nature of the dynamic system $\frac{df\left(t\right)}{dt}=f\left(y\right)$ without any model presumption. In fact, the model *f*(*y*) automatically emerges from the data set y. For simplicity, we shall be referring to the first derivative as “velocity” and the second derivative as “acceleration.” Using an LMM framework and the technique of BLUP, we can obtain the predicted platelet aggregation profiles. Note that based on the representation of *f*(*t*) from Equation (2), the first derivative (velocity) and the second derivative (acceleration) are as follows:

(6) $$\begin{array}{l} \frac{df\left(t\right)}{dt}=\;\overset{p-1}{\underset{j=1}{\sum }}j\beta_{j}t^{j}+\;\overset{K}{\underset{k=1}{\sum }}pu_{k}{\left(t-\;\kappa_{k}\right)}_{+}^{p-1}, \end{array}$$

(7) $$\begin{array}{l} \frac{d^{2}f\left(t\right)}{dt^{2}}=\;\overset{p-2}{\underset{j=2}{\sum }}{j\left(j-1\right)\beta }_{j}t^{j}+\;\overset{K}{\underset{k=1}{\sum }}{p\left(p-1\right)u}_{k}{\left(t-\;\kappa_{k}\right)}_{+}^{p-2}. \end{array}$$

A closer inspection of Equations (6) and (7) reveals that it is of the same functional form as that of Equation (2) and hence has a LMM representation. The estimated derivative curve is then obtained by replacing the parameters and the random effects in Equation (6) by estimated parameters $\hat{\beta }={\left({\hat{\beta }}_{0},\ldots ,{\hat{\beta }}_{p}\right)}^{\prime }$ and the predicted random effects of $û={\left({û}_{1},\;\ldots ,\;{û}_{K}\right)}^{\prime }$, respectively obtained from Equation (2). For a fixed dose of ADP, a way to describe the linear dynamical system would be to assume a differential equation of the form

(8) $$\begin{array}{l} \frac{\hat{d\;f\left(t\right)}}{dt}=\;\gamma_{0}+\;\gamma_{1}\hat{\;f\left(t\right)}+e_{t}, \end{array}$$

The form of the above equation suggests that regressing the predicted velocity function on the predicted platelet aggregation is equivalent to regressing the predicted velocity with the predicted fits where *e*_*t*_ is the random error assumed to be normally distributed with a zero mean and variance τ^2^, γ_0_ is the intercept and γ_1_ is the slope of the linear regression.

Dropping the assumption of linearity, we can extend the model in Equation (8) by assuming that the predicted derivative is a smooth function of the predicted platelet profile. In effect, we fit a model of the form,

(9) $$\begin{array}{l} \frac{\hat{d\;f\left(t\right)}}{dt}=\;\gamma_{0}+g\left(\hat{\;f\left(t\right)}\right)+e_{t}. \end{array}$$

i.e., we do not impose the constraint of a linear differential equation but assume that the velocity curve is related to the platelet aggregation curve via some smooth but otherwise unknown function *g*. As before, we model *g* using truncated basis functions in an LMM framework, thus allowing for a data driven estimation of the function *g*.

## Results

### Model Fitting and Estimation of Nanoparticle Effect

**Figure 1:**
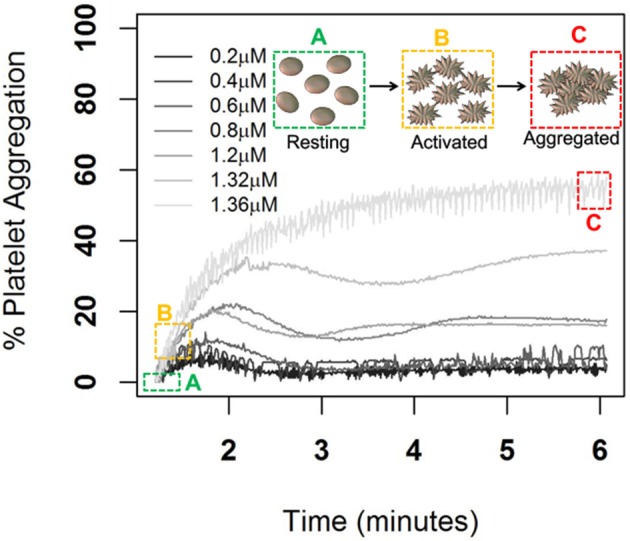
ADP induced Platelet aggregation profile of a normal healthy individual. Here aggregation was induced by different ADP concentrations from low to high (0.2, 0.4, 0.8, 1.2, 1.32, and 1.36 μm/ml) and respective schematic platelet response.

**Figure 2:**
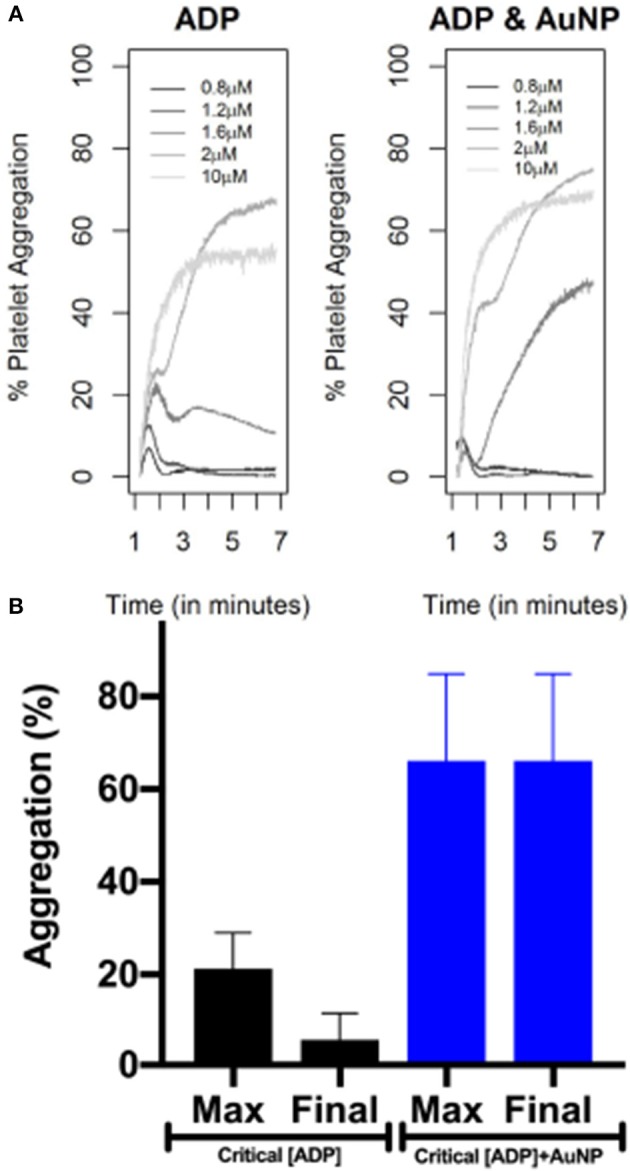
AuNP induced alteration of ADP induced platelet aggregation profile. Upper panel **(A)** is showing only ADP (of different concentrations) induced aggregation. Right panel is showing platelet aggregation in presence of 20 nm AuNP remaining other parameters exactly the same as left panel. The lower panel **(B)** is showing the change in maximum and final aggregation at the critical ADP concentration, while treated with AuNP (n = 10).

In Figure 1, we show the percentage of platelet aggregation for different concentrations of ADP. In general, the percentage of platelet aggregation increases with an increasing dose of ADP despite their different interim kinetic profiles. Figure 2 shows the effect of AuNP on the ADP induced platelet aggregation profile. Apparently, the profiles of the two groups (with and without AuNP) are similar with an exaggerated platelet aggregation activity in the group receiving nanoparticles. However, the kinetic pattern of individual aggregation induced by respective ADP concentration differs, while treated with AuNP. A critical ADP concentration observed as previously reported (Deb et al., 2011) where promotion of aggregation profile is profound in AuNP (Figure 2A) and showed a drastic increase in final aggregation from nearly 10% to more than 60% (Figure 2B).

**Figure 3:**
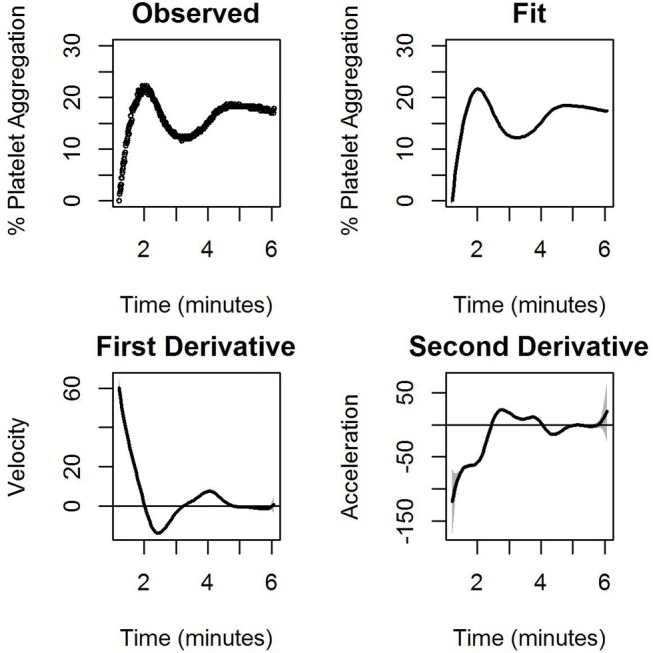
The observed percentage of platelet aggregation over time in response to administration of 1.32 μM of ADP together with model based estimates of the mean percentages, and point wise first and estimated second derivatives of the mean curve. The first derivative measures the velocity of aggregation while the second derivative measures the corresponding acceleration. A horizontal line indicating zero velocity and acceleration is also plotted for reference. The gray shaded region is the 95% confidence interval.

Figure 3 shows the observed and model-based estimates of percentage of the average platelet aggregation as a function of time (upper panel), while the bottom panel shows the observed model-based estimates of the pointwise first and second derivatives of the mean curve. Note that the latter two can be interpreted as the velocity and the acceleration curves. At any fixed point of time, the three curves measure respectively, the extent of platelet aggregation (after filtering out the noise), the instantaneous rate of change of platelet aggregation, and the instantaneous change in this rate. Thus, for example, a positive value of the velocity curve and a negative value of the acceleration curve denotes that aggregation increases at that point in time, but that there is a deceleration in the rate at which it increases. Figure 3 is based on data corresponding to the administration of 1.32 μM ADP. The detailed fits, means, and velocity for platelet aggregation obtained for all ADP concentrations for the first experiment have been elaborated on in Figure S1. Similar plots have been provided in the Supplementary Materials for the nanoparticle experiment on platelets. Figure S2 represents ADP induced platelet aggregation and Figure S3 represents the same experiment (platelets from same donor, same ADP concentration and execution of the experiment in the same time) with AuNP (20 nm, 40 μM).

Figure S1 shows that for all doses of ADP, there is an initial elevation in the graph (increase in aggregation), followed by fluctuations and plateau formations. With increasing ADP doses, increased steepness and height of the initial elevation, was also observed. After the initial elevation, we observed a reduction in the percentage of platelet aggregation, which was inversely proportional to the ADP dose, and in the final dose, no reduction was observed in the platelet aggregation profile. The derivative curves are approximately convex in nature, implying that platelet aggregation is increasing at a decreasing rate. The findings from the derivatives are consistent with our previous observation of initial aggregation followed by stabilization (Deb et al., 2011). All the derivatives dipped below zero within a time span of 2 to 3 min. With an increased dosage of agonist (ADP), the time to dip increases, signifying that the stabilization process is being deferred. Features related to acceleration are consistent with an initial upward movement of the platelet aggregation demonstrated by the upward trend in the acceleration curves, which corresponds to an initial increase in velocity.

**Figure 4:**
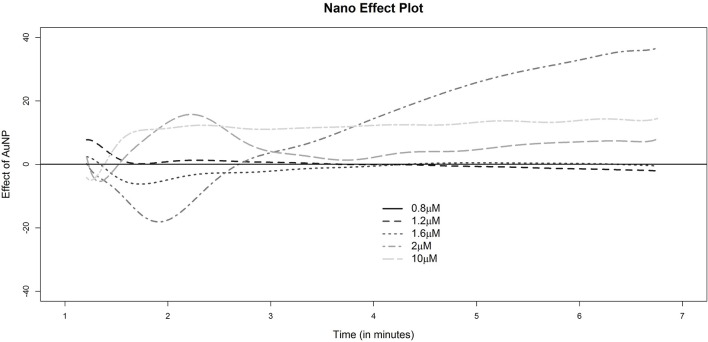
Nano effect plot. The height of any of the dotted lines at a fixed time point indicates the estimates excess (if positive) or deficit (if negative) platelet aggregation for the corresponding sample as indicated by the legend. The reference group for comparison corresponds to the sample with gold nanoparticles and a dose of 0.8 μM of ADP.A horizontal line at 0 is drawn for reference indicating no difference with this baseline group of 0.8 μM AuNP.

General findings from the nanoparticle perturbation experiments had largely similar features. However, with the addition of nanoparticles a striking difference in the curves was observed at low to medium ADP dose. Despite the fact that each curve showed similar initial features with a steep rise for the first three doses, the initial elevation induced by AuNP (Figure S3) was sharper than that for ADP alone (Figure S2). The elevation was so significant that for the medium dose level of ADP (1.6 μM), the shape of the curve was similar to that of ADP at a higher dose. Interestingly, a similar pattern was observed for the velocity curves. With the introduction of AuNP, the velocity of ADP induced aggregation at a dose of 1.6 μM, resembling that of higher doses of ADP alone. These findings are consistent with the fact that ADP concentration near critical concentrations can induce increased platelet aggregation similar to how higher concentrations of ADP induced aggregation, if AuNP is present in the medium (plasma). A detailed visual representation of how AuNP can affect platelet aggregation has been summarized as a “nano effect plot” in Figure 4.

### Differential Equation Model Results

We fit Equation (8) for all the seven dose levels of ADP for the first data. Table 1 shows the results of the model fitting. The γ_0_'s (slope) and γ_1_'s (intercept) are of the same sign and with an increase in dose, the γ_0_'s increase. Thus, with an increase in ADP concentration, the rate of platelet aggregation increases but with time the rate diminishes steadily as it tries to acquire a steady state. However, if we see the adjusted *R*^2^ (Adj. *R*^2^) values, which is an indicator of the model fit, it is clear that a linear differential equation is more appropriate for medium to higher doses of ADP.

**Table 1:** Table showing the estimated coefficients of Equation (8) for all the seven dose levels of ADP for the first experiment.

	**0.2 μg**	**0.4 μg**	**0.6 μg**	**0.8 μg**	**1.2 μg**	**1.32 μg**	**1.36 μg**
γ_0_ (SE)	**1.49** (0.62)	**8.34** (1.68)	**8.84** (0.98)	**46.41** (2.46)	**66.13**(2.55)	**68.5** (1.88)	**80.10** (0.75)
γ_1_(SE)	−0.24 (0.17)	–**1.31** (0.29)	–**1.28** (0.15)	–**2.62** (0.15)	–**4.02** (0.16)	–**2.02** (0.06)	–**1.5** (0.02)
Adj. *R*^2^	0	0.04	0.13	0.39	0.56	0.69	0.95

*The coefficients in bold have p < 0.05. Here, γ_0_ and γ_1_ are the respective slope and intercept of the fitted linear regression. The numbers in bracket indicate the Standard Errors (SE). The Adj. R^2^ value is an indicator of the model fit with higher values indicating a better fit*.

**Figure 5:**
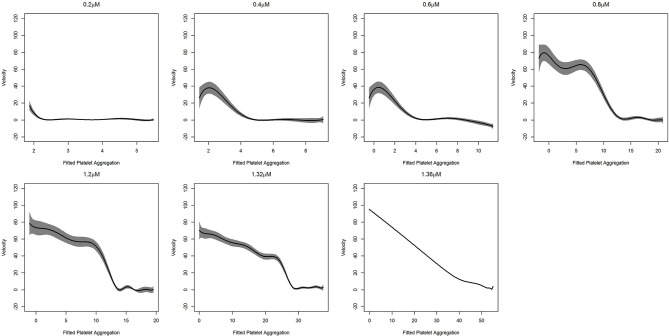
Fitted curve for agonist experiment. Here result of the fit obtained from (9). The predicted velocity is plotted at fitted values of the platelet aggregation. The solid line represents the fit while the shaded portion is the computed 95%confidence interval. To facilitate comparison, all the plots have the same vertical axis limits. A solid line at zero velocity is also drawn for reference.

**Figure 6:**
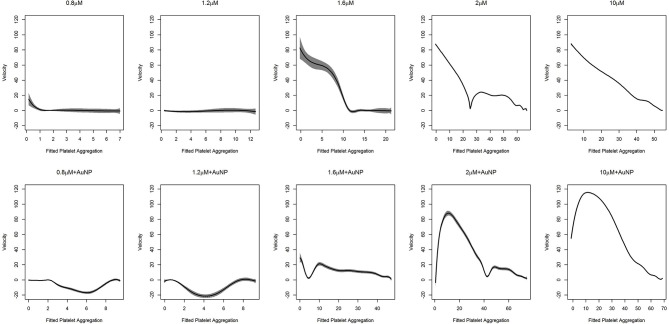
Fitted curve for nanoparticle experiment. Result of the fit obtained from (9) has plotted here. The predicted velocity is plotted at fitted values of the platelet aggregation. The solid line represents the fit while the shaded portion is the computed 95% confidence interval. The first row of plots represents the fits for different levels of ADP only. The second row represents the fits for ADP with the fixed concentration of nanoparticles (AuNP 40 nm). To facilitate comparison, all the plots have the same vertical axis limits.

In the context of platelet aggregation, a linear dynamic system does not seem to be a perfect model, particularly for low doses of ADP, and hence we fit the more general regression model in Equation (9). The results of the fit, with the confidence interval for the two datasets, are shown in Figures 5, 6.

Once the dynamic system is defined, we can then subject the same to test the stability analysis that reflects the sensitivity of the system with respect to perturbation around the steady state.

As was deduced, for higher doses of ADP, the dynamic system behaves linearly. We clearly observed that the rate of platelet aggregation decreased with a decrease in platelet aggregation percentage. In short, with an increased dose of ADP, an increasing initial rate of platelet aggregation was observed, which was also accompanied by a steeper decrease in the rate of platelet aggregation. The kinetics of platelet aggregation is consistent with the findings presented in Figure 5 and in the upper row of Figure 6. However, with the addition of nanoparticles, a change in these patterns was observed (lower row of plots in Figure 6). At low doses of ADP, the rate of aggregation decreases from zero, but, after a certain amount of platelet aggregation, the rate shoots up and tries to stabilize at zero velocity. We also observed this feature at the medium ADP concentration, albeit the initial velocity was not zero. The rate of platelet aggregation formation starts from a moderate value, decreases rapidly, shoots up, and then decreases comparatively slowly to reach zero velocity. The trajectory for this can be viewed as a combination of two features, where initially the rate goes down and then rises, after which it starts to drop. The initial feature is similar to that of the behavior of platelet aggregation for lower doses of ADP along with AuNP. Platelet aggregation at higher doses of ADP in combination with AuNP, demonstrated the exaggerated version of the second feature for the medium dose level. Contrary to its counterpart, with the introduction of AuNP, the platelet aggregation sharply increases initially and then starts to decrease sharply to reach zero velocity.

## Discussion

In the present study, we had three main objectives: (a) to construct a platelet aggregation model using aggregometric experimental data, (b) to use model parameters to describe the aggregation/de-aggregation phenomenon in activated platelets, and (c) to assess the response, to reference agonist ADP while perturbing with gold nanoparticles.

Born's platelet aggregometry (Paniccia et al., 2015) (chrono-log) based platelet aggregation test (Figure S8) is an easy, smart and most widely used gold standard technique to measure platelet functions in diseases, such as ACS, and bleeding disorders (Kottke-Marchant and Corcoran, 2002; Hayward, 2008; Guha et al., 2009a,b; Hayward et al., 2009) to name a few. In addition, this technique allows the measurement of aggregation in plasma or whole blood and hence, the native physiological conditions are maintained, unlike other molecular techniques like flow cytometry (De Cuyper et al., 2013) and western blot (Reid et al., 1990) where a buffer is used. Despite several advantages of optical aggregometry, only a few parameters, like final platelet aggregation, average slope, lag time, and the area under the curve, are being encased as the output result from the aggregometry study, which are insufficient for the interpretation of the whole physiological kinetics behind the complete aggregation process (Figures 1, 2 and Figure S7). Therefore, this real-time aggregometry kinetics has the potential to probe the dynamics of platelet functions, but owing to the lack of advanced analytical tools, the complete dataset does not contain any information about changes of platelet physiology during aggregation and the correlation of these changes with the normal physiological system. On the other hand, existing molecular techniques can track the changes within platelet micro-environments; however, they are only capable of delivering limited information about the inter-platelet physiology or dynamics during aggregation. A complete understanding of hemostasis is not possible until we have a clear idea about platelet aggregation kinetics (inter-platelet interaction) during aggregation. We believe that the present report will help further understand the pathological condition, and consequently will help clinicians in better managing patients during disease onset and progression. To the best of our knowledge, there is almost no biological tool that explores platelet kinetics using inter-platelet interactions as its key component. Therefore, in the present study, we have extracted detailed features of platelet aggregation (from optical aggregometry) under agonist (in this case ADP) and nanoparticle (AuNP) perturbation conditions, using a linear mixed model in functional data analysis to better understand the fundamental platelet aggregation process.

The LMM formulation has several advantages for this purpose. First, it can be fitted using standard software such as the nlme package of R (Pinheiro et al., 2019). Second, the use of a linear mixed model framework allows the use of established techniques such as BLUP for estimation of the platelet aggregation profiles, as well as the estimation of the instantaneous velocities and accelerations (Figure 3). The first and higher order derivatives proved to be a useful pointer to the shape of a curve. In biomedical settings, this is useful as it reveals a clearer idea about how the response changes with changes in the continuous covariates.

We found that the velocity of aggregation always decreases with time in the presence of a higher agonist (ADP) concentration (last panel in Figures S1, S2), with a sharp and steep aggregation pattern. In contrast, low to medium agonist concentrations (0.2–1.2 μM in Figure S1) could increase the velocity in some cases when there is de-aggregation. This increasing pattern of velocity is presumably the signature of de-aggregation. The physiological significance of increasing and decreasing velocity patterns could be explained as follows: a) at higher ADP concentrations, the platelets are incorporated into the aggregation process over time and hence available resting platelets become reduced over time, resulting in a decrease in velocity; b) on the other hand, in the presence of low to medium ADP concentrations, platelets oscillated between aggregation and de-aggregation states as lower ADP concentrations are insufficient for triggering an intense aggregation. Therefore, the loosely bound aggregates or micro aggregates (local minima in the curves) become destabilized (de-aggregation) after a certain time, which results in increased velocity. In this context, another important perspective should also be mentioned; platelets contain granules (alpha and dense) with many small molecules and agonists and ADP is one of the main agonists in the alpha granule (Flaumenhaft, 2003). Upon activation, platelets release their granular content and ADP released from the granules plays a crucial role in further maintaining the aggregation process via the activation of neighboring platelets. Platelets have two ADP receptors, P2Y1 and P2Y12, where P2Y1 requires a very low ADP concentration, although its activation is insufficient for the formation of stable platelet aggregates, whereas P2Y12 activates at higher ADP concentrations and is responsible for stable aggregate formation (Jagroop et al., 2003). Therefore, in lower ADP concentrations (as described in Figures S1, S2), P2Y1 possibly becomes active and triggers mild platelet activation which helps form unstable aggregates or microaggregates that destabilize (de-aggregation) over time. Interestingly, sometimes this mild activation can stimulate the release of sufficient ADP from the granules itself, leading to an increased global ADP concentration, which triggers the next phase of aggregation by activating the neighboring platelets. Thus, this is to reconfirm that platelets are going through oscillations of aggregation and de-aggregation behavior as described in Figures S1, S2. The crucial final fate of activated platelets (de-aggregation, mild aggregation, or hyper-aggregation) is dependent on the balance between global effective concentrations of ADP and the fraction of activated purinergic receptors (P2Y1 or P2Y12) at that time. In a complex biological system, the presence of two distinct stages is a typical signature of a bistable system. Such bistability is exhibited in the case of platelet aggregation as demonstrated in Figure 5. Except at a high ADP concentration (1.36 μM), the platelet aggregation process has two steps. First, there was a staircase phenomenon where the rate of change of thrombus size was oscillating between first order kinetics and zero order kinetics, which corresponded to the generation of a number of unstable local minima. After reaching a critical mass, the velocity underwent a sharp drop and stabilized at zero, which resulted in the formation of a stable aggregate, which represents a global minimum in the curve. This bistability is possibly due to the interplay between two purinergic receptors and the resultant release of ADP stored in granules. In the presence of a high ADP concentration, only one purinergic receptor (P2Y12) plays a major role in platelet aggregation and thus the aggregation process becomes first order.

**Figure 7:**
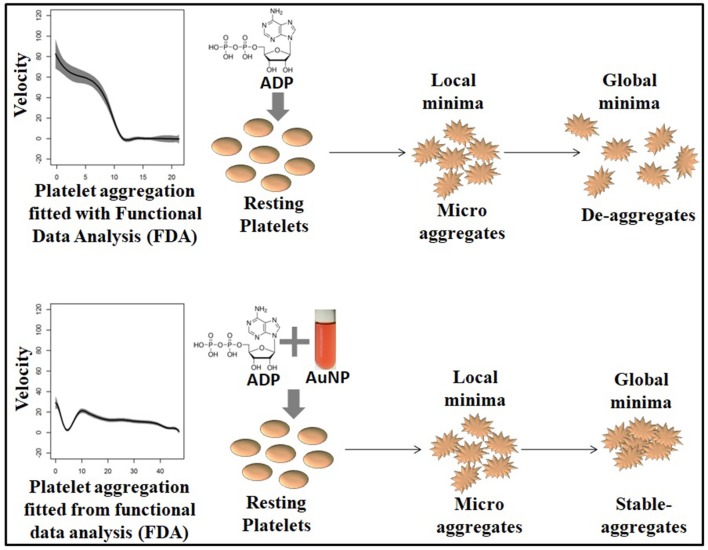
Schematic interpretation of gold nanoparticle (AuNP) induced bistability.

In a previous study, we showed that 40 μM of AuNPs with a hydrodynamic diameter of 20 nm (Figure S5) is the most potent perturbing agent of platelet stability in the presence of a critical concentration of ADP (Figure 2, Figures S6, S7) (Deb et al., 2011). In the present study, where AuNPs of the same diameter and concentration induced perturbation of platelet aggregation, three remarkable features were uncovered. First, it has been derived that average fluctuation of velocity and in some cases the rate of change of platelet aggregation were much higher compared to those of only ADP induced aggregation (Figures 2, 3, 6, Figures S2, S3). Second, during aggregation, the local minima (pseudo-steady point) and global minimum (steady point) were higher in case of AuNP induced aggregation (Figure 6). This indicates that the masses of initial microaggregates and final aggregates were higher in the presence of AuNP. A summary of AuNP induced platelet aggregation is illustrated in Figure 7. Lastly, we have shown that the rate of platelet formation was only reversed (positive) for the AuNP, at the critical ADP concentration where a de-aggregation to aggregation jump was observed (Figure S4). This is consistent with our earlier claims about the critical ADP concentration dependency of nanoparticle effects on platelets (Deb et al., 2011). We have developed a “nano effect plot” to easily track and monitor this critical ADP concentration pertaining to the nanoform effects on functional platelet aggregation profiles (Figure 4). This will certainly give us a rational choice about their risk-benefits profile, while also developing nanoformulations for biomedical applications.

## Conclusions

It can be deduced that platelet aggregation is a bistable system, where the transition from one phase to another is dependent on the ADP concentration and purinergic receptor interplay. This interplay is greatly influenced by the nanoparticles in an autocatalytic way, resulting in a higher aggregate mass both at the pseudo-stable point and stable point. This effect was most pronounced near the critical concentration of ADP, where a phase shift from the de-aggregated to the aggregated state occurred. Further studies could explore the initial pseudo-stable point, as this metastable state is very susceptible to any stimuli, including nanoparticle perturbations and could be an interesting target for disease diagnosis and/or nanoparticle toxicity assessment.

## Data Availability

The raw data supporting the conclusions of this manuscript will be made available by the authors, without undue reservation, to any qualified researcher.

## Ethics Statement

We have ethical approval (No/NMC/4655 dated 27/08/2016) and consent of participant of this study.

## Author Contributions

SB contributed to the functional data analysis and writing the primary draft of the manuscripts. AD revised the manuscript. BG, SS, and HP provided intellectual contributions to the manuscript. HP designed, wrote, and revised the manuscript. SD contributed to the concept and design of the work, performed experiments, and wrote the manuscript. MA helped ploting the results and revising the manuscript.

## Conflict of Interest Statement

SB was employed by company GlaxoSmithKline Asia Pvt. Ltd. The remaining authors declare that the research was conducted in the absence of any commercial or financial relationships that could be construed as a potential conflict of interest.
