# Supplementary material for: Probing ADP Induced Aggregation Kinetics During Platelet-Nanoparticle Interactions: Functional Dynamics Analysis to Rationalize Safety and Benefits
Source: Front Bioeng Biotechnol. 2019 Jul 18;7:163. doi: 10.3389/fbioe.2019.00163 (PMC6657536; doi:10.3389/fbioe.2019.00163)
Supplement: Supplementary file 1 [file Data_Sheet_1.doc]

Supplementary Materials:


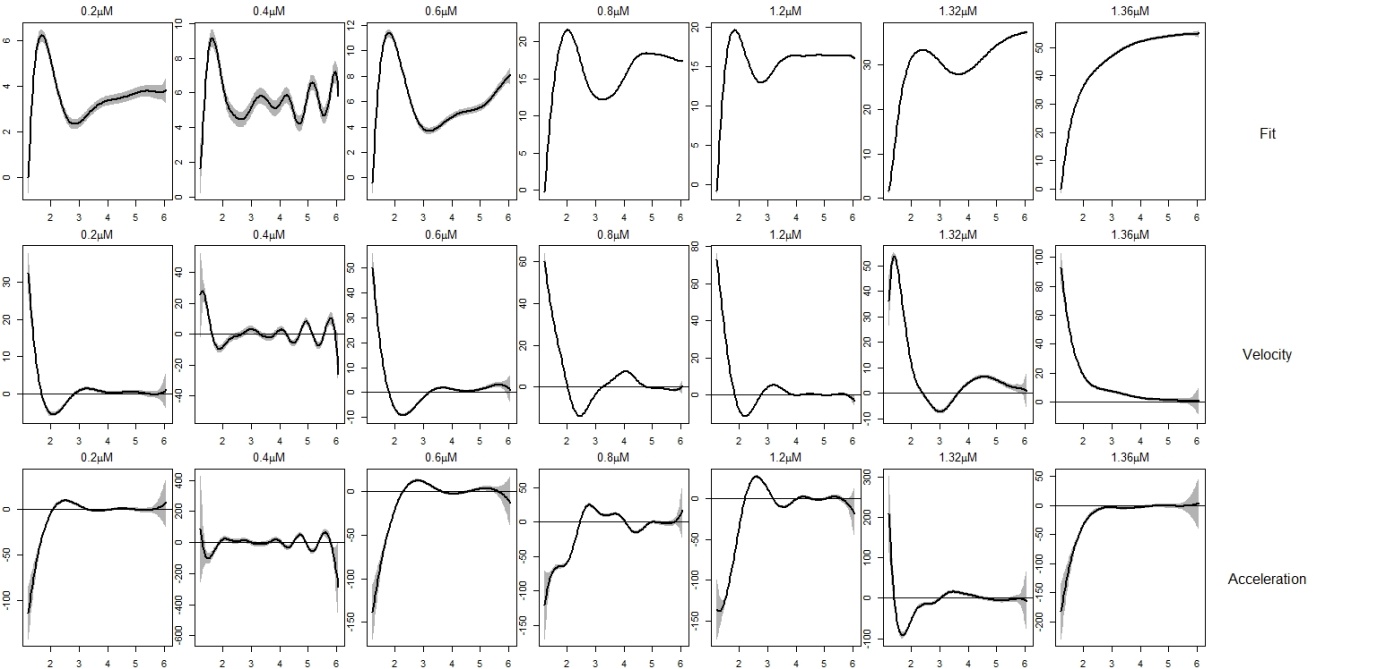


Figure 1: The estimated fits, first derivative (velocity) and second derivative (acceleration) of the data from agonist experiment. The grey shaded region is the 95% confidence interval. The horizontal axis is time in minutes. A horizontal line passing through zero, indicating zero velocity and acceleration is drawn for all velocity and acceleration plots.


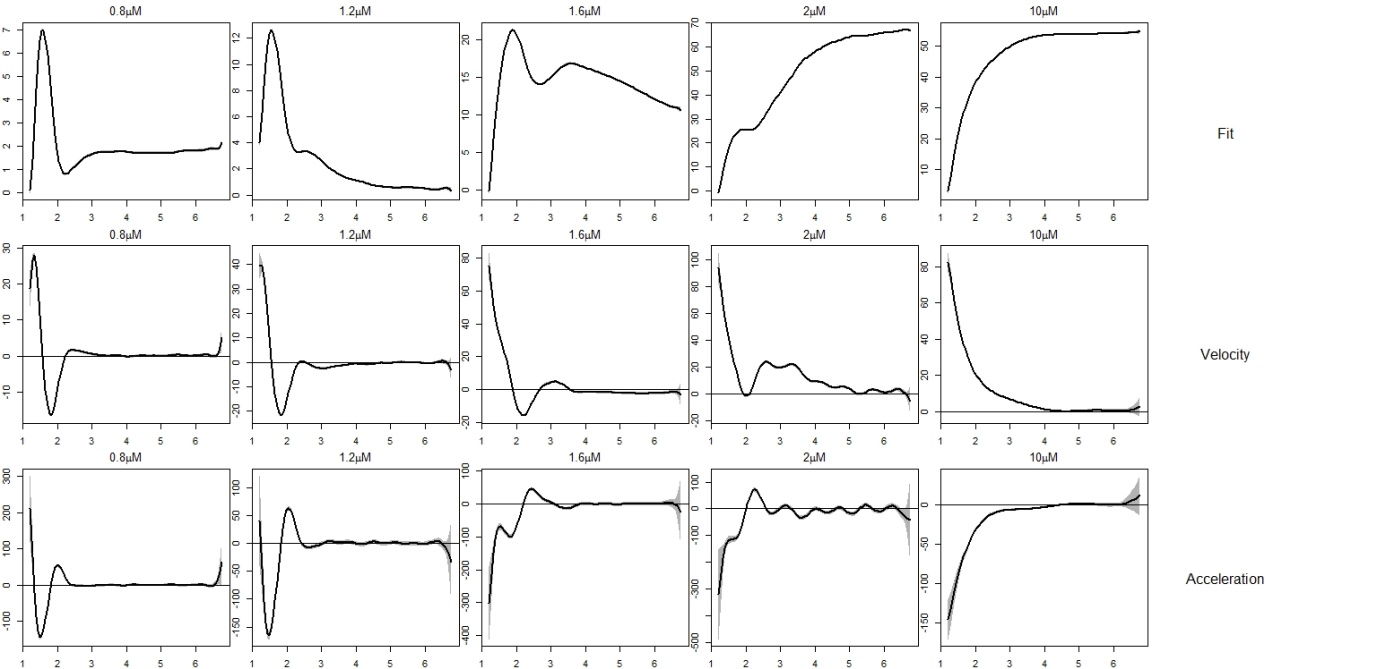


Figure 2: Estimated fits, first derivative (velocity) and second derivative (acceleration) for different doses of ADP for the nanoparticle experiment. A horizontal line passing through zero, indicating zero velocity and acceleration is drawn for all velocity and acceleration plots. The horizontal axis is time (in minutes).


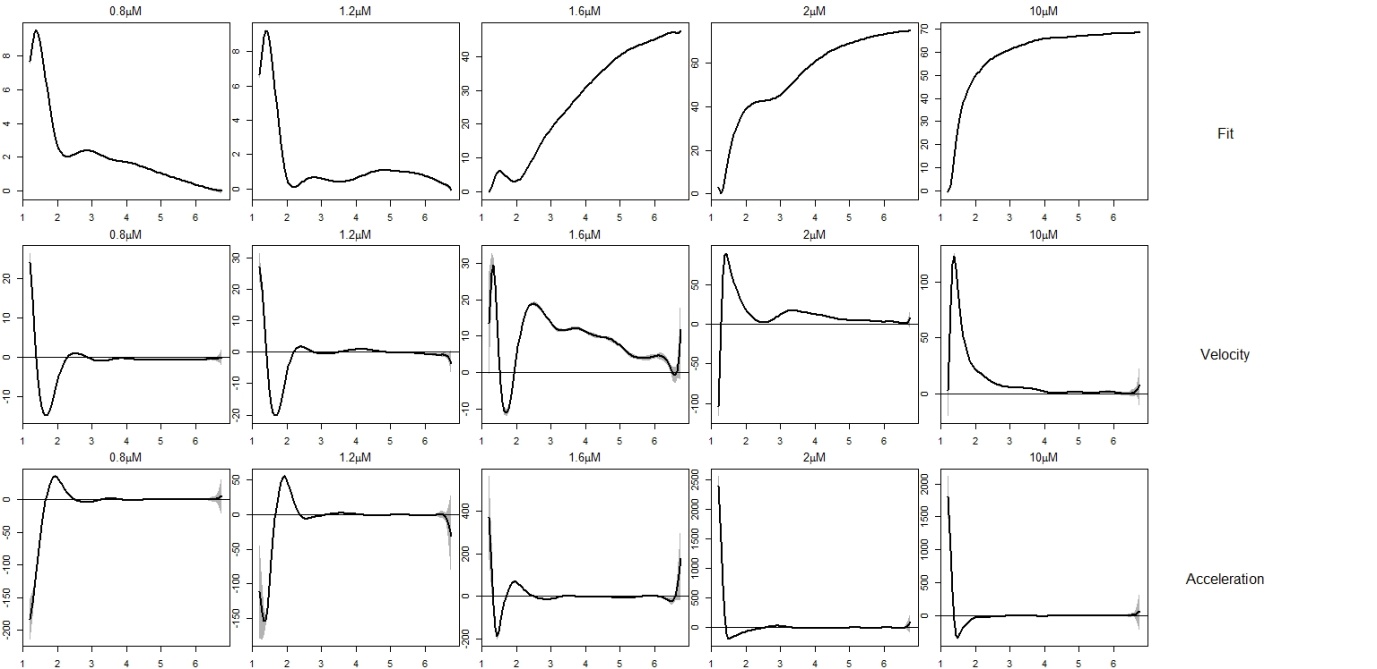


Figure 3: Estimated fits, first derivative (velocity) and second derivative (acceleration) for different doses of ADP and AuNP for the nanoparticle experiment. A horizontal line passing through zero indicating zero velocity and acceleration is drawn for all velocity and acceleration plots. The horizontal axis represents as time in minute.


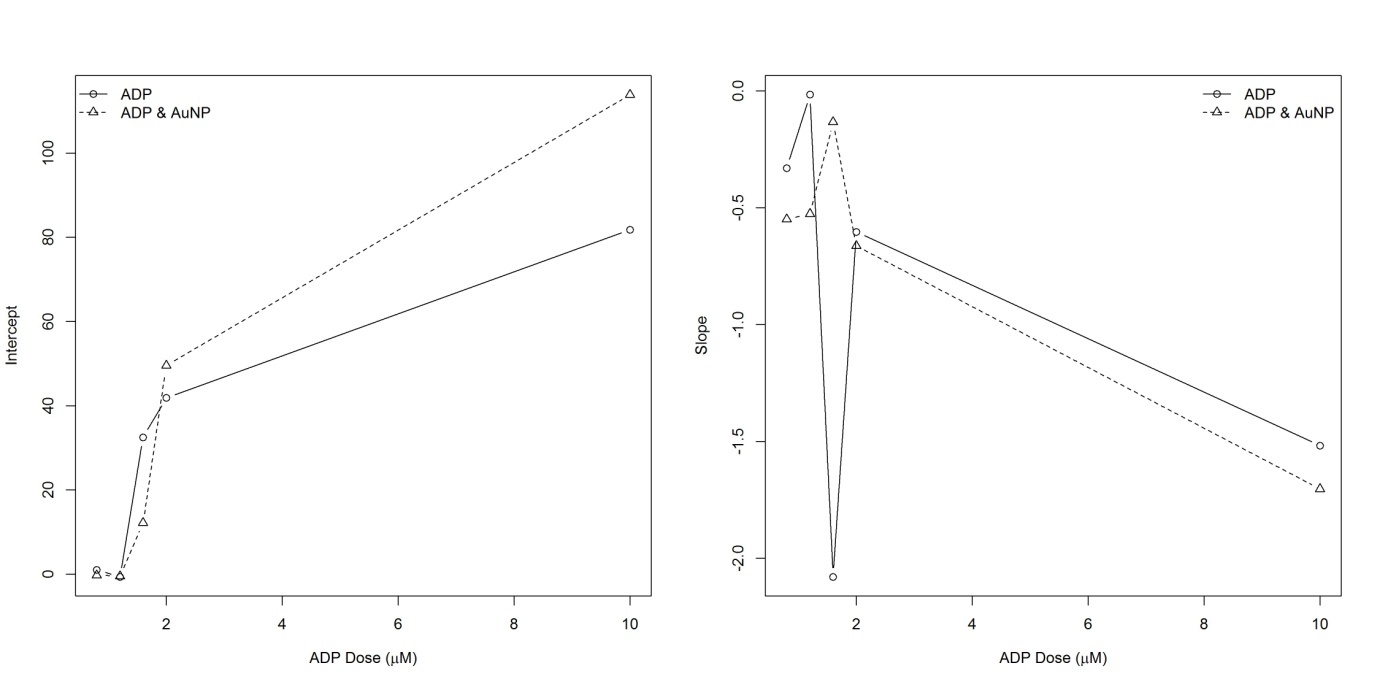


Figure 4: Behaviour of intercept and slope of first order derivative in presence and absence of AuNP when platelets are treated with varying doses of ADP.

**Gold nanoparticle Preparation and Characterization**:

Gold nanoparticle (AuNP) preparation: AuNPs were prepared by the citrate reduction of HAuCl4. Briefly an aqueous solution of HAuCl4 (496 μM, 6.25 ml) was stirred continuously by automatic stirrer, in a boiling condition; then 380 μl of, 20 mM of tri-sodium citrate stock solution was added to it quickly, resulting in an orange coloured solution. After the colour change, the solution was refluxed for 10 min and was allowed to cool at room temperature. Synthesised AuNP then characterised through Malvern NanoZS (for hydrodynamic radius and surface zeta potential) and Photon Technology International Inc (for spectral analysis).


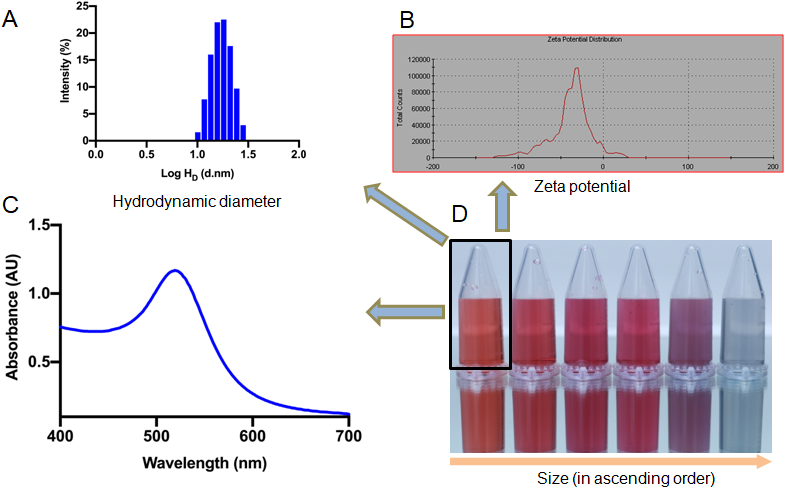


Figure5: Nanosystem: A series of gold nanoparticles have been synthesized using wet chemical citrate based reduction method for the present purpose. The optimized particle has been used for this study has 20nm hydrodynamic diameter with -34mV zeta potential measured through Dynamic Light Scattering (DLS) (A-B). The surface plasmon resonance is around 520nm with high stability in blood plasma (C-D).

The washed platelet suspension was incubated with AuNP (20nm 40µM) at 37 °C for 5 min with stirring (1000 rpm) condition and then subjected to activate with ADP at 10 μM. Platelets were then washed with HEPES buffer and incubated for 60 min at 4°C in dark, with 2 μL FITC-conjugated antibody (PAC1) for GpIIbIIIa. 2% paraformaldehyde then added to the platelet suspension in equal amount and incubated at dark for 30 min. After washing with HEPEs buffer platelets were then finally suspended in phosphate buffer saline (PBS) and analyzed in a Becton Dickinson FACS Calibur flow cytometer.


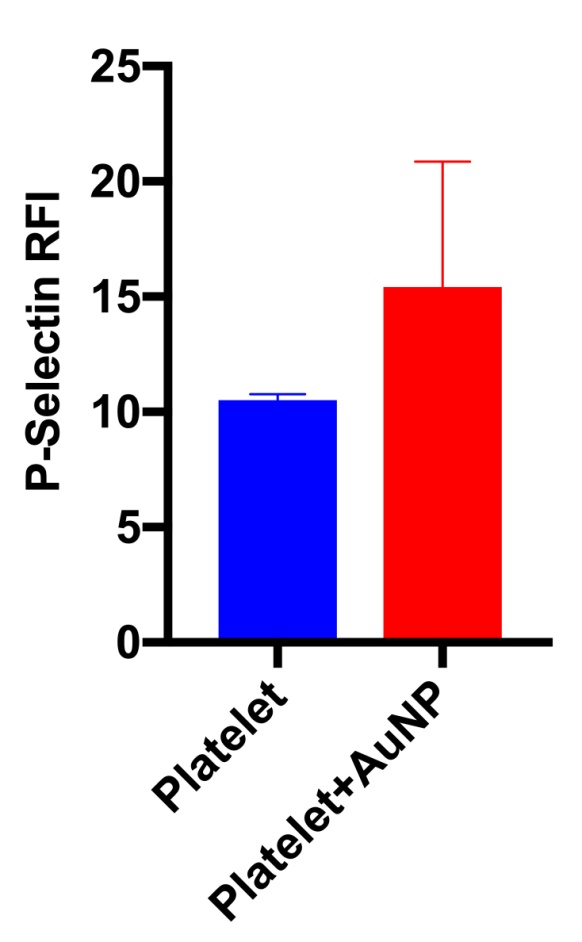


Figure 6: represents exocytosis of alpha granules (detected as P-selectin expression) in the presence of AuNP detected through flow cytometer (n=10).

Here Platelet Rich Plasma (PRP) was activated with ADP 0.4µM alone or in presence of AuNP (20nm 40µM). To get a microscopic view of platelet aggregates in different time points (resting or zero time, initial aggregation or local minima and final aggregation or global minima), aggregation reaction was stopped using platelet fixative agent (containing 0.4 glutaraldehyde and 4% paraformaldehyde in PBS buffer). Fixed samples was then examined under light microscope (Nikon eclipse Ti)


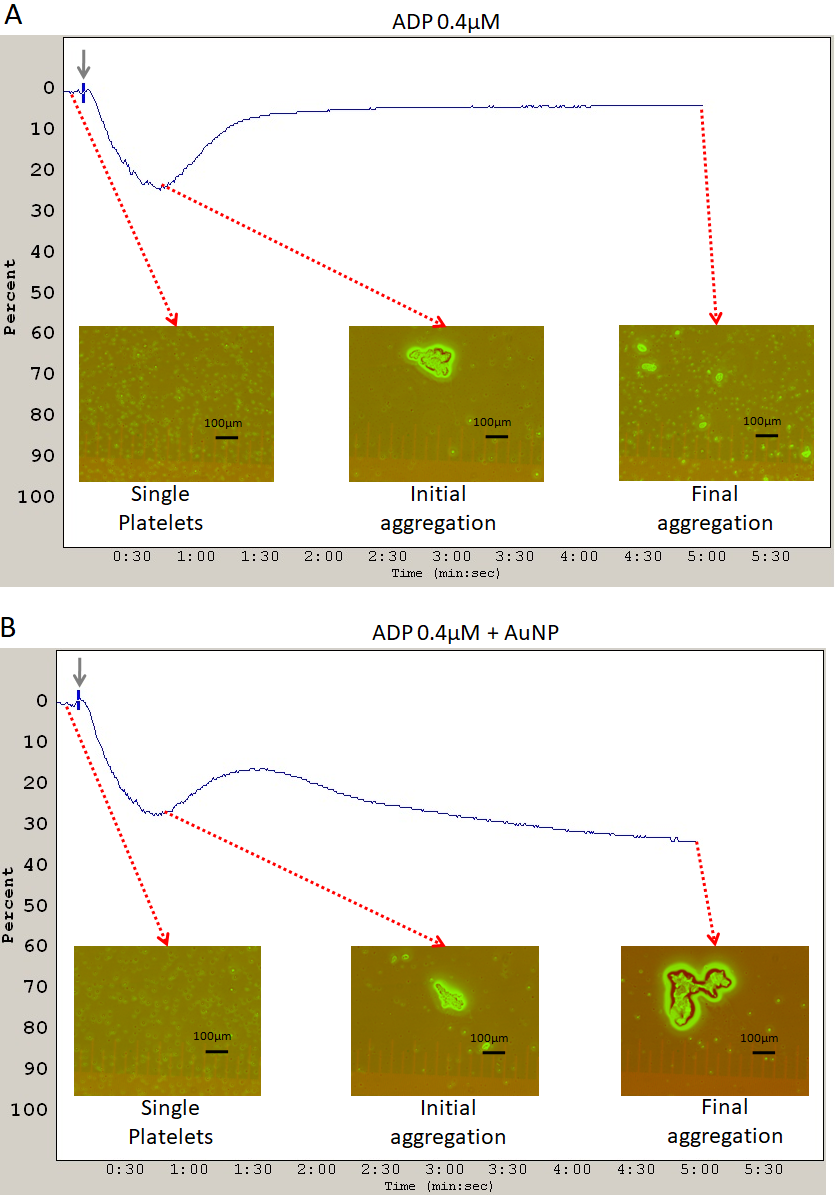


Figure 7: represents microscopic view of platelet aggregates in different time point of platelet aggregation. The small gray down ward arrow represents here the agonist (ADP0.4µM) addition time.


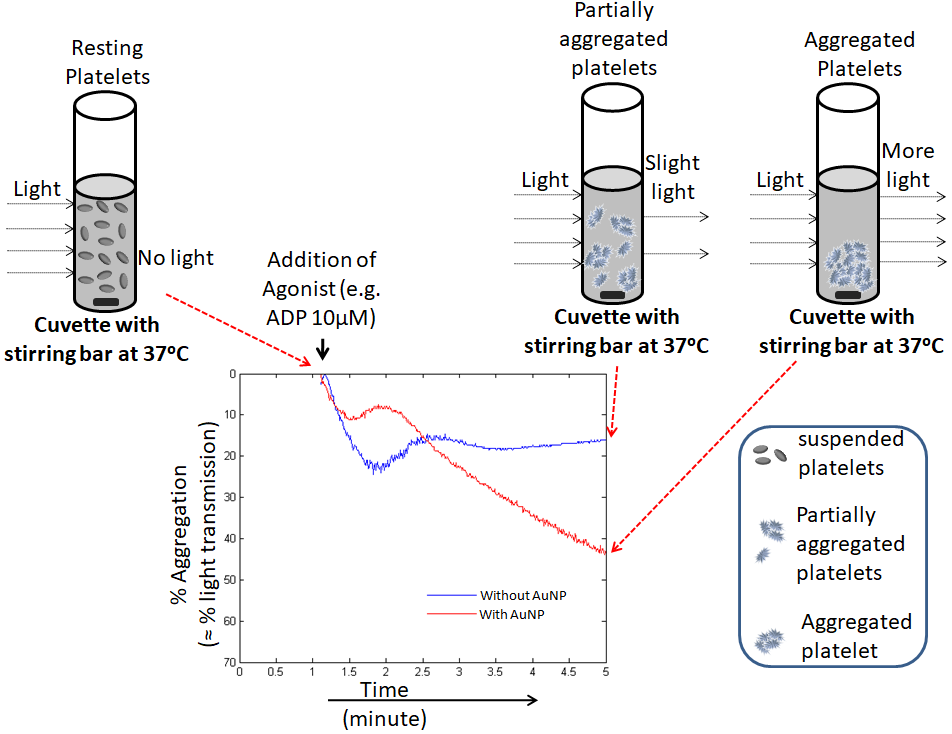


Figure 8: Schematic diagram of principal of platelet aggregation through optical aggregometry.
